# Development of rapid antigen test prototype for detection of SARS-CoV-2 in saliva samples

**DOI:** 10.48101/ujms.v127.8207

**Published:** 2022-02-25

**Authors:** Agnija Kivrane, Viktorija Igumnova, Elza Elizabete Liepina, Dace Skrastina, Ainars Leonciks, Zanna Rudevica, Svjatoslavs Kistkins, Aigars Reinis, Anna Zilde, Andris Kazaks, Renate Ranka

**Affiliations:** a Latvian Biomedical Research and Study centre, Ratsupites Street 1, k–1, Riga, LV1067, Latvia; b Riga Stradins University, Dzirciema Street 16, Riga, LV1007, Latvia; c Pauls Stradins Clinical University Hospital, Pilsonu street 13, Riga, LV1002, Latvia

**Keywords:** Lateral flow assay, ELISA, COVID-19, polyclonal antibodies, antigen test, point-of-care testing

## Abstract

**Background:**

The development of easy-to-perform diagnostic methods is highly important for detecting current coronavirus disease (COVID-19). This pilot study aimed at developing a lateral flow assay (LFA)-based test prototype to detect severe acute respiratory syndrome coronavirus 2 (SARS-CoV-2) virus in saliva samples.

**Methods:**

Mice were immunized using the recombinant receptor-binding domain (rRBD) of SARS-CoV-2 virus spike protein. The combinations of the obtained mouse anti-receptor-binding domain (RBD) polyclonal antibodies (PAbs) and several commercial antibodies directed against the SARS-CoV-2 spike protein were used for enzyme-linked immunosorbent assay (ELISA) to select antibody pairs for LFA. The antibody pairs were tested in a LFA format using saliva samples from individuals with early SARS-CoV-2 infection (*n* = 9). The diagnostic performance of the developed LFA was evaluated using saliva samples from hospitalized COVID-19 patients (*n* = 111); the median time from the onset of symptoms to sample collection was 10 days (0–24 days, interquartile range (IQR): 7–13). The reverse transcription-polymerase chain reaction (rRT-PCR) was used as a reference method.

**Results:**

Based on ELISA and preliminary LFA results, a combination of mouse anti-RBD PAbs (capture antibody) and rabbit anti-spike PAbs (detection antibody) was chosen for clinical analysis of sample. When compared with rRT-PCR results, LFA exhibited 26.5% sensitivity, 58.1% specificity, 50.0% positive prediction value (PPV), 33.3% negative prediction value (NPV), and 38.7% diagnostic accuracy. However, there was a reasonable improvement in assay specificity (85.7%) and PPV (91.7%) when samples were stratified based on the sampling time.

**Conclusion:**

The developed LFA assay demonstrated a potential of SARS-CoV-2 detection in saliva samples. Further technical assay improvements should be made to enhance diagnostic performance followed by a validation study in a larger cohort of both asymptomatic and symptomatic patients in the early stage of infection.

## Introduction

Severe acute respiratory syndrome coronavirus-2, namely SARS-CoV-2, is a novel strain of coronavirus belonging to the genus *Betacoronavirus*, which primarily causes interstitial pneumonia; however, in severe cases it may also affect other organ systems (1, 2). Started as a local outbreak in December 2019, it has given rise to the global pandemic with over 213 million cases of infection and 4.5 million deaths reported so far (3). Timely set diagnosis allows for prevention of further spreading of the infection, thus increasing the demand for high-throughput diagnostic testing.

World Health Organization (WHO) and leading health authorities have formulated various recommendations for effective management of the SARS-CoV-2 crisis, including guidance for diagnostics (4, 5). The real-time reverse transcription-polymerase chain reaction (rRT-PCR) is the primary diagnostic method to detect viral ribonucleic acid (RNA) in nasopharyngeal and oropharyngeal swabs (5). The rRT-PCR features high sensitivity, specificity, and diagnostic accuracy. Also, viral RNA can be detected in various biological matrices over a broad range of viral load, that is, in the asymptomatic phase, during the ongoing infection, and months after the initial infection (6–8). Nevertheless, the overall turnaround time might last from hours to days because the sampling should be performed by trained medical personnel, and the analysis is carried out in specially equipped laboratories by laboratory technicians following stringent biosafety precautions.

The use of lateral flow assay (LFA)-based rapid antigen tests is restricted to application in outbreak investigation and mass screening programs when the rRT-PCR capacity might be insufficient (4, 9). Besides, a confirmatory rRT-PCR test is often required to verify the result of the rapid test (4). In addition to the limited diagnostic performance of the rapid tests, antigen-based tests authorized by the Member states of the European Union mostly employ nasal or oropharyngeal swabs as testing material (10). The benefits of such tests are cost-effectiveness, simple workflow, and much shorter turnaround time, as the result is determined visually within 30 min. Thus, it is important to continue the research and development process to improve the clinical performance of rapid tests and to implement point-of-care testing for timely identification and isolation of infected persons. Moreover, pioneer studies exploring the presence of virus in biological fluids and experience from the incorporation of antigen-based tests in SARS-CoV-2 mass screening programs have confirmed that saliva as an easy-collectable testing material allows for overcoming practical issues related to sample collection (11–13).

This study aimed to develop a LFA-based test prototype directed against SARS-CoV-2 antigens in saliva samples. The clinical performance of the proposed assay was evaluated by analyzing saliva samples of hospitalized coronavirus disease (COVID-19) patients.

## Materials and methods

### Clinical samples

Saliva samples from volunteers with confirmed SARS-CoV-2 infection were collected. They were within the first 5 days after the onset of symptoms (*n* = 7). Healthy study participants (*n* = 4) comprised the training set that was used for assay development. Samples were self-collected by the patient in a sterile collection tube without any preservatives added. At the time of collection, all patients had no symptoms or mild symptoms, and were recovering at home.

The clinical performance of the proposed LFA prototype was assessed by analysis of a test set. The test set consisted of saliva samples (*n* = 113) from SARS-CoV-2 virus-positive patients admitted to the Pauls Stradiņš Clinical University Hospital, Department of Quarantine (No. 80) and Department of Pulmonology and Internal medicine (No. 14a) in December, 2020 – January, 2021. Samples were self-collected by the patient or with the assistance of medical personnel in a sterile collection tube without any preservatives added.

Sample handling procedures and experimental work were carried out in a biosafety level-2 (BSL2) environment. The saliva samples were homogenized by adding 500 μL of 1× phosphate-buffered saline (PBS) directly into the collection tube and thoroughly vortexing for 2 min. The obtained samples were stored at –70ºC before analysis.

### Production of recombinant SARS-CoV-2 protein

The particular region encoding the receptor-binding domain (RBD) of SARS-CoV-2 spike protein with the N-terminal 6-His tag was amplified and cloned into the pPICZα vector (Invitrogen, Carlsbad, CA, USA) behind the α-factor secretion signal using *Xho*I and *Not*I restriction sites, thus restoring the Kex2 signal cleavage site. Plasmid inserts were verified by Sanger sequencing. Valid constructs were linearized at the *Mss*I site and transformed by electroporation into the *Pichia pastoris* X-33 strain. Mut^+^ transformants were obtained on agar containing yeast peptone dextrose (YPD) plates with zeocin (400 μg/mL). The cultivation of the selected clone was performed in Buffered Glycerol Complex Medium (BMGY) medium at 24°C with an agitation rate of 250 rpm for 24 h. Then, cultivation was continued for 2 more days with the daily addition of 1% methanol to the cell medium. After the final incubation stage, the cell medium was briefly centrifuged to pellet cells. The obtained supernatant was mixed with buffer solution (50 mM Tris–HCl (pH 8.0) and 300 mM NaCl) and loaded on the His Trap FF crude column (GE Healthcare, Uppsala, Sweden) for affinity purification. The bound protein fraction was eluted using a linear imidazole gradient and subsequently concentrated on the Amicon 10-kDa centrifugal filter unit (Millipore, Burlington, MA, USA) to an approximate volume of 2.0 mL. The additional purification step involved passing the obtained concentrate through the size-exclusion Superdex 200 column (Sigma-Aldrich, St. Louis, MO, USA) in the PBS. The target protein fraction (25 kDa) was identified by sodium dodecyl sulfate-polyacrylamide gel electrophoresis (SDS-PAGE) and Coomassie staining to be further used for antibody production and immunoassays.

### Production of mouse PABs

Six to eight weeks old female Bagg Albino (BALB)/c mice (*n* = 10) were received from the University of Tartu, Laboratory Animal centre and held at the Latvian Biomedical Research and Study Centre. The experimental procedure was approved by the Latvian Animal Protection Ethics Committee and the Latvian Food and Veterinary Service (permission No. 89, received 12.07.2017.). The mice were subcutaneously immunized three times at 2-week intervals (Days 0, 14, and 28) with recombinant receptor-binding domain (rRBD) protein at a dose of 25 μg per mouse. On Day 0, before immunization, blood samples were collected to obtain blank serum, which is used as a negative control. The antigen was diluted in PBS and complete Freund’s adjuvant (CFA, SigmaAldrich, St. Louis, MO, USA) (1:1, v/v) for the first injection, but for the remaining two injections – in PBS and incomplete Freund’s adjuvant (IFA, Sigma-Aldrich, St. Louis, MO, USA) (1:1, v/v). The control group (*n* = 2) was subject to the same immunization scheme as the experimental group but received PBS-adjuvant injections without the antigen added. On Day 42, animals were bled to obtain serum samples containing anti-rRBD PABs (anti-rRBD). The end-point titer was determined by direct ELISA and defined as the highest serum dilution that yielded three times greater absorbance when compared with the control group.

### LFA test prototype development

#### Antibody screening using sandwich ELISA

The assay was performed to test the ability of antibody to bind the SARS-CoV-2 antigen. Antibodies used in this study were targeting epitopes of the SARS-CoV-2 spike protein. The antibodies were as follows: mouse anti-rRBD PABs (obtained in this study, designated as antibodies A), mouse anti-spike S1 MABs (antibodies B) (Sino Biological, Beijing, China), mouse anti-RBD MABs (antibodies C) (Arigobio, Hsinchu, Taiwan), and rabbit anti-spike (antibodies D) and anti-spike/RBD PABs (antibodies E) (Sino Biological, Beijing, China). The recombinant SARS-CoV-2 spike protein S1/S2 (rS1/S2 protein; Sino Biological, Beijing, China) was used as an antigen in this assay.

A flat-bottom 96-well microtiter plate (Nunc MaxiSorp, Thermo Fisher Scientific, USA) was coated with 50 μL of capture antibodies (1:1,000 diluted in 0.1 M bicarbonate–carbonate buffer, pH 9.6) and then incubated overnight at 4°C. Subsequently, the plate was washed twice with 1× PBS (pH = 7.4), and 1% bovine serum albumin (BSA) solution in 1×PBS was filled into each well to block the remaining protein binding sites and incubated for another 24 h. The blocking solution was then discarded, and 50 μL of 1.25 μg/mL rS1/S2 protein solution in 1× PBS were added. The incubation continued for 90 min at 37°C. Negative control (without antigen and detection antibodies) and blank sample (without antigen) were introduced in each plate. After four times of washing with 0.5% BSA solution in 1× PBS, 50 μL of secondary antibodies (1:1,000 diluted in 0.5% BSA solution in 1× PBS) were added and then incubated for 90 min at room temperature. Then, the plate was washed four times with 0.5% BSA solution in 1× PBS, then 50 μL of HRP-conjugated sheep anti-mouse IgG or donkey anti-rabbit IgG antibodies (ImmunoResearch Laboratories, Inc., West Grove, PA, USA) diluted (1:5,000) in 0.5% BSA solution in 1× PBS were added to each well, and incubated for 60 min at room temperature. Repeatedly, the plate was washed four times with 0.5% BSA solution in 1× PBS. The enzymatic reaction was performed by the addition of 100 μL of 3,3’,5,5’-tetramethylbenzidine solution (Sigma-Aldrich, St. Louis, MO, USA) to each well, which was stopped by the addition of 50 μL of 1 M sulfuric acid. The absorbance of the yellow colored reaction product was spectrophotometrically measured at 450 nm using a plate reader.

#### LFA procedure

In the LFA format, the Universal LFA kit (Abcam, UK) was used to evaluate the most promising antibody combinations selected for SARS-CoV-2 antigen detection. The capture antibody labelling with Lighting-Link^®^ allows the formed antibody–antigen complex to be covalently bonded to the nitrocellulose membrane of the test strip. However, detection antibody labelling with colloidal gold nanoparticles enables visualization of the capture antibody-antigen-detection antibody sandwich at the test line. At the control line, *the result appears due to the interaction between streptavidin immobilized on the strip streptavidin and gold-biotin conjugate added to the reaction mixture.*

Before conjugation, antibodies were pre-treated with Antibody Purification Kit Protein A and Antibody Purification Kit-Nanoparticles (Abcam, UK) following the manufacturer’s protocol to remove excess buffer constituents that could potentially interfere with conjugation and affect the conjugate quality. Accordingly, capture antibodies were subjected to conjugation with Lighting-Link^®^ Ulfa-Tag conjugation kit, but detection antibodies for the conjugation with 40 nm gold particles using Gold Conjugation kit according to manufacturer’s instructions.

Before analysis, conjugates were diluted with 1× Running Buffer solution containing 0.1% BSA (1×RB) added as the blocking agent to a final concentration of 50 mg/mL and 8 optical density units (OD) for capture and detection antibodies, respectively. Similarly, the supplied 40 mm gold-biotin conjugate (10 OD) was diluted to reach a final concentration of 1 OD.

Description of the assay procedure: To the 75μL of the saliva sample, 5 μL of each diluted reagent were added, briefly vortexed, and incubated for 5 min at room temperature. The reaction mixture (80 μL) was transferred to the separate well in a 96-well plate; the test strip was immersed into the corresponding well and left for another 10–20 min. If sample flow was interrupted, the 1× RB (20–70 μL) was added to the sample pad to reduce excessive viscosity and facilitate sample flow. The test results were simultaneously evaluated by two independent investigators. If the control line was not detected after the time provided, the test was considered as failed, and samples were reanalyzed. The sample was classified as positive only if the test line appeared in the presence of the control line.

The saliva samples from the training set were included in each analytical batch serving as positive and negative controls.

### Real-time RT-PCR

#### Viral RNA isolation from clinical samples

Initially, an aliquot of a saliva sample (100 μL) was subjected to virus inactivation for 5 min at 95°C. RNA was isolated with TRI Reagent^®^ (Sigma-Aldrich, St. Louis, MO, USA) according to the manufacturer’s protocol for RNA isolation from the cell suspension. SARS-CoV-2 positive saliva samples from the training set were introduced into each test sample batch for the quality control of RNA isolation and RT-PCR. The obtained RNA pellet was then reconstituted in 60 μL of nuclease-free water and used for further analysis.

#### Clinical sample analysis

The SARS-CoV-2 E gene-based real-time RT-PCR assay (rRT-PCR) was adapted from the WHO-approved protocol (14) for SARS-CoV-2 detection in clinical samples. This assay served as a reference method for qualitative and quantitative evaluation of saliva samples.

First, complementary DNA (cDNA) synthesis was carried out in a separate step using High-Capacity cDNA Reverse Transcription kit (Applied Biosystems, USA) and SARS-CoV-2 E gene-specific reverse primer (Table 1). A volume of 10 μL isolated RNA was used for reverse transcription. The rRT-PCR analysis was performed on QuantStudio™ 7 Flex Real-Time PCR System (Thermo Fisher Scientific, USA) using TaqMan Fast Advanced Master Mix (Thermo Fisher Scientific, USA) and a set of E gene-specific primers and probe (Table 1). The thermal cycling conditions used: uracil–DNA glycosylase (UNG) pre-treatment at 50°C for 2 min, initial denaturation at 95°C for 20 s, 40 cycles of 95°C for 3 s, 60°C for 30 s (data acquisition). The reaction volumes were 20 and 4 μL of that comprised synthesized cDNA.

**Table 1:** Primers and probe* targeting the SARS-CoV-2 virus E gene used in the rRT-PCR analysis.

Name	Sequence (5′–3′)
E_Sarbeco_F1	ACAGGTACGTTAATAGTTAATAGCGT
E_Sarbeco_R2	ATATTGCAGCAGTACGCACACA
E_Sarbeco_P1	FAM-ACACTAGCCATCCTTACTGCGCTTCG-BHQ-1

* Published by Corman and colleagues (14).

Six calibration standards of artificial SARS-CoV-2 E gene plasmids were prepared in nuclease-free water and analyzed in duplicate in each analytical run along with study samples. The six-point calibration curve was constructed by plotting the C_t_ value against the E gene copy number in the calibration standard, which was used to calculate the viral load in the study samples. The limit of detection was 1.5 × 10^3^ copies/mL, with the quantification range from 3 × 10^3^ to 3 × 10^8^ copies/mL.

Data acquisition and analysis were performed with QuantStudio™ Real-Time PCR Software (v1.3; Thermo Fisher Scientific, USA).

### Statistical analysis

Statistical analysis of data was performed in MedCalc Software (v19.7; Ostend, Belgium). The developed LFA assay was evaluated in terms of clinical sensitivity and specificity, positive predictive value (PPV), negative predictive value (NPV), and diagnostic accuracy.

## Results

### Sandwich ELISA

In total, 12 different tests in the sandwich ELISA format were performed to evaluate possible antibody combinations (Table 2). Irrespective of antibody combination, all antibody pairs exhibited the binding ability to the recombinant SARS-CoV-2 antigen. Signal variability between antibody pairs can be explained by the competitive binding of antibodies to a single epitope or the absence of the specific binding sites on the surface of the rS1/S2 antigen. The antibody A obtained in this study by mouse immunization with rRBD and commercial antibodies showed comparable reactivity toward the rS1/S2 antigen. To avoid inconsistency between both methods, matching antibody pairs were further tested in LFA format.

**Table 2:** Performance assessment of the antibody combinations using sandwich ELISA.

Capture antibody	Detection antibody	Measured absorption (A_450 nm_)
A	D	1.662
	E	1.243
B	D	0.967
	E	0.276
C	D	0.878
	E	0.353
D	A	0.938
	B	0.238
	C	0.219
E	A	1.206
	B	0.165
	C	0.167

* Antibodies: A – mouse anti-rRBD PABs (obtained in this study), B – mouse anti-spike S1 MABs (Sino Biological, China), C – mouse anti-RBD MABs (Arigobio, Taiwan), D – rabbit anti-spike PABs (Sino Biological China), E – rabbit anti-spike/RBD PABs (Sino Biological, China). The plate was subsequently coated with capture and detection antibodies diluted to 1:1,000 in an appropriate diluent. As an antigen, the recombinant rS1/S2 protein (Sino Biological, China) was used at a concentration of 1.25 μg/mL. The assay results were determined spectrophotometrically at λ = 450 nm.

### Establishment of the LFA protocol for clinical sample analysis

Based on the ELISA results, the A, B, and C antibodies were selected as capture antibodies, whereas D and E antibodies were selected as detection antibodies to be tested on saliva samples from the training set. For that purpose, a series of preliminary experiments with rS1/S2 protein spiked in SARS-CoV-2 negative saliva samples at a concentration of 1 μg/mL were performed to determine working concentrations of the antibody conjugates and sample handling technique (data not shown).

**Figure 1:**
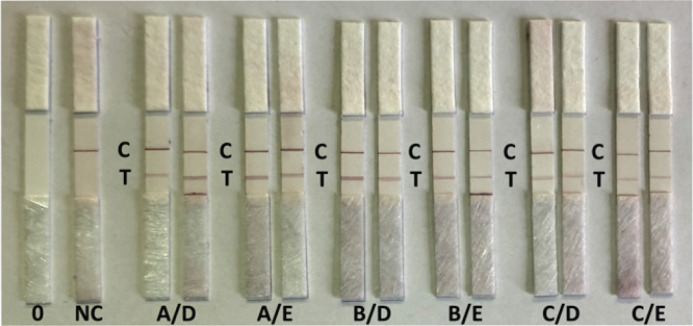
LFA rapid antigen test results for six antibody pairs, each test was run in duplicate. 0: unused strip; NC: negative control; C: control line; T: test line.

Further tests with the SARS-CoV-2 positive saliva samples from the training set confirmed that all six possible antibody combinations could be used to detect SARS-CoV-2 in salivary samples (Figure 1). Moreover, antibody pairs B/E and C/E (capture antibody/detection antibody) that produced a weak signal in the ELISA test (A_450 nm_ < 0.5) demonstrated acceptable performance in an LFA format. Antibody E directed against the spike/RBD protein shared common antibody binding sites with the B and E antibodies on the rS1/S2 protein.

In the assay development, it was necessary to maintain assay specificity and avoid false-negative results due to possible competitive antigen binding. Therefore, it was decided to select an antibody pair targeting two different antigens. As the antibody pair A/D with custom-made capture antibody A demonstrated the same performance as the antibody pair (C/D) composed of commercial antibodies, further LFA tests were conducted with A/D antibody pair directed against SARS-CoV-2 virus spike protein RBD domain and spike protein.

### Characterization of study samples: test set

From the 113 saliva samples received from Pauls Stradiņš Clinical University Hospital, two did not meet the eligibility criteria and were excluded from the study. One sample lacked signed informed consent from the study participant, but the other had visible traces of blood.

The final test set consisted of saliva samples from 111 patients with the laboratory-confirmed SARS-CoV-2 infection (Table 3). Median time between laboratory-confirmed diagnosis and sample collection was 6 days (0–21 days, IQR 3–10 days). Test sample set structure is shown in Supplementary Fig. 1.

**Table 3:** Test sample set structure and time-based classification of saliva samples.

Features			Test set	Subgroups	
Time between laboratory-confirmed diagnosis and sample collection	Presence of COVID-19 symptoms	Time between the symptom onset and sample collection	Total (*n* = 111)	Group A* (*n* = 88)	Group B** (*n* = 38)
≤7 days (*n* = 72)	Symptomatic (*n* = 58)	≤7 days (*n* = 24)	X	X	X
		>7 days (*n* = 34)	X	X	
	Asymptomatic and unknown (*n* = 14)	Unknown (*n* = 14)	X		X
>7 days (*n* = 39)	Symptomatic (*n* = 30)	≤7 days (*n* = 0)			
		>7 days (*n* = 30)	X	X	
	Asymptomatic and unknown (*n* = 9)	Unknown (*n* = 9)	X		

* Group A comprised 88 saliva samples from symptomatic SARS-CoV-2 patients.

** Group B comprised 38 saliva samples that were collected ≤7 days after the first symptom onset (*n* = 24), samples from asymptomatic patients and those for whom the time of symptom onset could not be specified, but the time between laboratory-confirmed diagnosis and sample collection was ≤7 days (*n* = 14).

Based on the available clinical information (date of laboratory-confirmed diagnosis and self-reported date of symptom onset), the samples were stratified into subgroups. Group A comprised saliva samples (*n* = 88) from SARS-CoV-2 patients with symptomatic form of the disease and a known date of symptom onset (Table 3). For group A, the median time between the symptom onset and sample collection was 10 days (0–24 days, IQR: 7–13) (Supplementary Fig. 1). Group B included saliva samples that were collected ≤7 days after the onset of first symptom (*n* = 24), samples from asymptomatic patients, and those for whom time of symptom onset could not be specified, but the time between laboratory-confirmed diagnosis and sample collection did not exceed 7 days (*n* = 14) (Table 3).

**Figure 2:**
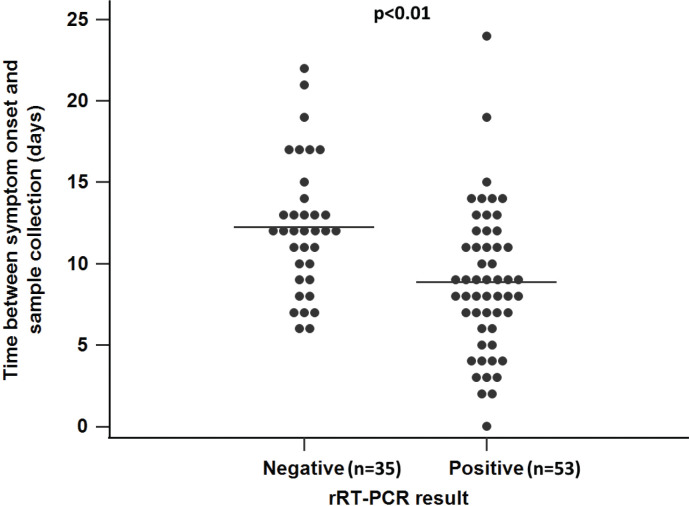
Distribution of the rRT-PCR test results depending on the time interval between the symptom onset and saliva sample collection. The interval of time elapsing between the onset of symptoms and sample collection was significantly shorter in the rRT-PCR-positive sample group (median 9 vs. 12 days, Mann–Whitney, *P* < 0.01).

The rRT-PCR was considered as the reference method used for sample qualitative and quantitative characterization. The assay results were expressed as binary (positive/negative) and continuous variables (viral load, log_10_ copies/mL). Although all study participants had previously laboratory-confirmed COVID-19 diagnosis, during sample collection, viral RNA was detected only in 61.3% of the samples, and the median viral load for the RNA-positive samples was 1 × 10^4.1^ gene copies/mL (IQR: 1×10^3.0^–1×10^5.0^) (Table 4). Accordingly, when samples were stratified based on the symptom onset time and/or time from laboratory-confirmed diagnosis, 60.2% in Group A and 81.6% in Group B were SARS-CoV-2 RNA-positive; the median viral loads for the RNA-positive samples in Group A and Group B were 1×10^4.0^ gene copies/mL (IQR: 1 × 10^3.0^–1 × 10^4.6^) and 1 × 10^4.3^ gene copies/mL (IQR: 1 × 10^3.3^–1×10^6.0^), respectively (Table 4). In Group A, the viral load tended to negatively correlate with the onset of symptoms (Spearman’s *r* = –0.42, *P* < 0.01). Also, in symptomatic patients, the median time between the symptom onset and sample collection was significantly shorter in the group of rRT-PCR positive samples compared with those that were rRT-PCR negative (median 9 vs. median 12 days, Mann–Whitney, *P* < 0.01) (Figure 2).

**Table 4:** Overview of the rRT-PCR and LFA test results.

	Group A*(*n* = 88)	Group B**(*n* = 38)	Test sample set (*n* = 111)
Sample classification based on the rRT-PCR result at the moment of sampling			
rRT-PCR-positive, n (%)	53 (60.2%)	31 (81.6%)	68 (61.3%)
rRT-PCR-negative, n (%)	35 (39.8%)	7 (18.4%)	43 (38.7%)
Viral load in the rRT-PCR-positive samples			
Median, copies/mL (IQR)	1×10^4.0^ (1×10^3.0^–1×10^4.6^)	1×10^4.3^ (1×10^3.3^–1×10^6.0^)	1×10^4.1^ (1×10^3.0^–1×10^5.0^)
Sample classification based on the LFA test result			
LFA-positive, n (%)	25 (28.4%)	12 (31.6%)	36 (32.4%)
LFA-negative, n (%)	63 (71.6%)	26 (68.4%)	75 (67.6%)

IQR: xxx.

* Group A included saliva samples from symptomatic SARS-CoV-2 patients.

** Group B included saliva samples that were collected ≤7 days after first symptom onset (*n* = 24), and samples from asymptomatic patients and those for whom the time of symptom onset could not be specified, but the time between laboratory-confirmed diagnosis and sample collection did not exceed 7 days (*n* = 14).

### LFA results and clinical performance

Following the developed LFA protocol, the characterized saliva samples from the test set (*n* = 111) were evaluated for the presence of SARS-CoV-2 antigens. The LFA result was positive for 36 samples (32.4%) but negative for 75 samples (67.5%) (Table 4).

**Figure 3:**
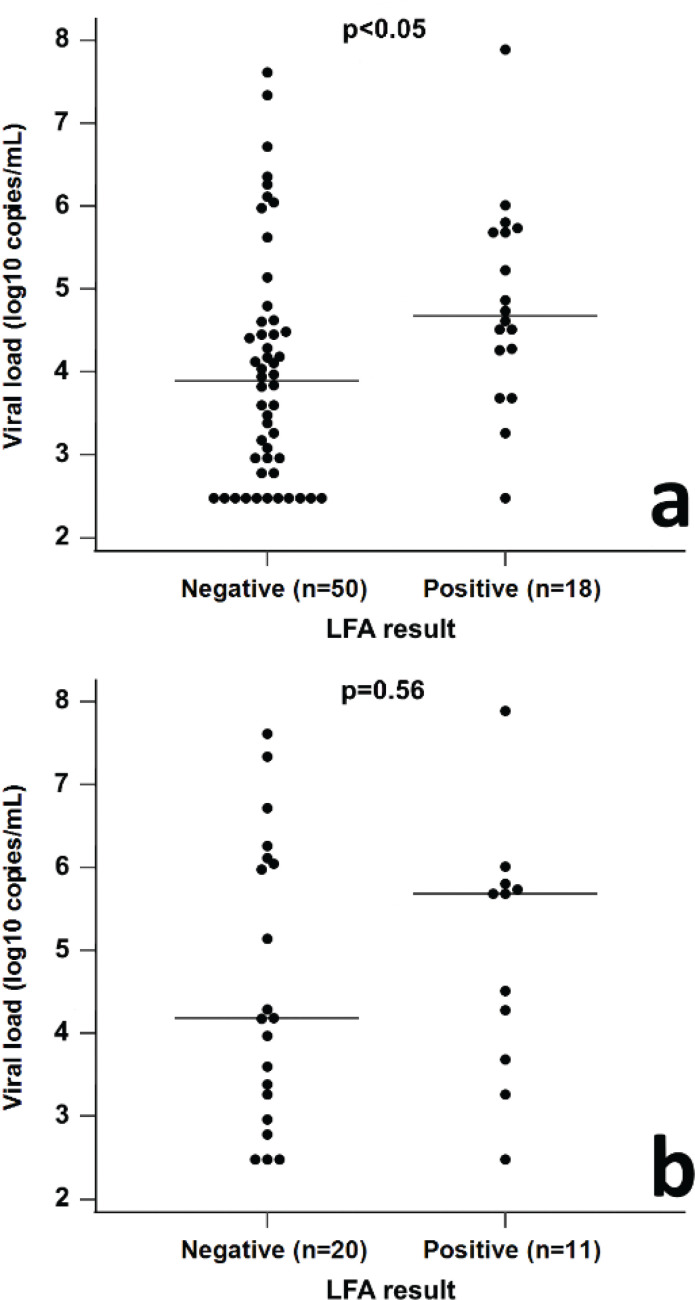
Distribution of the LFA rapid antigen test results of true-positive (rRT-PCR-positive) saliva samples depending on the viral load. (a) The difference in viral load was significant between LFA-positive and LFA-negative sample groups (median 1 × 10^4.7^ vs. median 1 × 10^3.9^ copies/mL, Mann–Whitney, *P* < 0.05) (*n* = 68). (b) Results of subgroup analysis that comprised only true-positive samples collected ≤7 days after the symptom onset and saliva samples for whom the time between laboratory-confirmed diagnosis and sample collection was ≤7 days (*n* = 31). For these samples, the differences between LFA-positive and LFA-negative sample groups with respect to median viral load were not statistically significant (Mann–Whitney, *P* = 0.56).

Among the true-positive samples (*n* = 68), the viral load was significantly higher in the LFA-positive sample group (median 1 × 10^4.7^ vs. median 1 × 10^3.9^ copies/mL, Mann–Whitney, *P* < 0.05) (Figure 3a). The same tendency was observed; however, the statistical significance disappeared when the analysis was limited to the true-positive samples in group B, that is, saliva samples that were collected ≤7 days after the onset of first symptom, and samples from asymptomatic patients and those for whom the time of symptom onset could not be specified, but the time between laboratory-confirmed diagnosis and sample collection did not exceed 7 days (*n* = 31) (Mann–Whitney, *P* = 0.56) (Figure 3b).

Next, diagnostic performance of the LFA test was evaluated using data from the whole test sample set as well as using data from Group A and Group B separately. The obtained results are summarized in Table 5, and contingency tables are presented in Supplementary Table 1. Compared with rRT-PCR results, the sensitivity and specificity of the proposed LFA assay in the test sample set were 26.5 and 58.1%, respectively. The probability of detecting SARS-CoV-2 in the saliva of an infected person or PPV was 50.0%, whereas the probability of a true-negative result or NPV was 33.3%, with the diagnostic accuracy 38.7%. The strength of the agreement with the reference rRT-PCR method was considered weak (Cohen’s *k* < 0.2).

**Table 5:** Diagnostic performance assessment of the developed LFA rapid antigen test using saliva samples from patients with previously laboratory-confirmed SARS-CoV-2 infection.

Parameter	Assay result, % (95% CI)		
	Test sample set (*n* = 111)	Group A (*n* = 88)*	Group B (*n* = 38)**
Sensitivity	26.5 (16.5–38.6)	20.8 (10.8–34.1)	35.5 (19.2–54.6)
Specificity	58.1 (42.1–73.0)	60.0 (42.1–76.1)	85.7 (42.1–99.6)
PPV	50.0 (37.0–63.0)	44.0 (28.8–60.4)	91.7 (62.8–98.6)
NPV	33.3 (27.2–40.1)	33.3 (27.0–40.4)	23.1 (16.7–30.9)
Diagnostic accuracy	38.7 (29.6–48.5)	36.4 (26.4–47.3)	44.7 (28.6–61.7)

CI: confidence interval.

* Group A included saliva samples from symptomatic SARS-CoV-2 patients.

** Group B included saliva samples that were collected ≤7 days after the first symptom onset (*n* = 24), and samples from asymptomatic patients and those for whom the time of symptom onset could not be specified, but the time between laboratory-confirmed diagnosis and sample collection did not exceed 7 days (*n* = 14).

The diagnostic performance of the LFA assay remained low when evaluated specifically in the samples from symptomatic COVID-19 patients (Group A) (Table 5). To reduce time bias, the diagnostic performance analysis was performed using data from Group B saliva samples. Overall, the exclusion of samples that were collected >7 days after the first symptom onset and/or >7 days after laboratory-confirmed diagnosis led to an increase in all parameters with major improvements in specificity (85.7%) and PPV (91.7%).

## Discussion

Rapid and accurate diagnostic testing is one of the prerequisites for successful disease management. Current opinions on rapid antigen-based test utility in clinical practice are still controversial due to their variable sensitivity that highly depends on the sample quality and viral load (15). Moreover, several recent studies have reported that even authorized antigen-based tests can experience a weaker diagnostic performance than expected (16). The cost-effectiveness, simple handling, and test result within 30 min are the key considerations to employ rapid-antigen tests. For example, in outbreak investigation and control or to support mass screening programs in schools, prisons, and healthcare centers. Hence, this study was conducted responding to the global pandemic and increasing demand for high-throughput testing. Instead of commonly used nasopharyngeal and oropharyngeal swabs, we offered self-collectable saliva samples as testing material for rapid detection of SARS-CoV-2 in the LFA-based format. The antibody pair consisting of mouse anti-rRBD PAbs and commercial rabbit anti-spike PAbs was incorporated into the LFA-based assay to target different epitopes on the virus surface, and the clinical performance of the proposed assay was evaluated by analysis of saliva samples from hospitalized COVID-19 patients.

Several published studies suggest that saliva samples could be a reliable alternative for SARS-CoV-2 diagnostics. Diagnostic assays using saliva as a testing material have proven to have at least similar sensitivity when compared with nasopharyngeal swabs (17, 18), and results on LFA-based rapid test targeting SARS-CoV-2 antigens in saliva samples are already available (19).

The viral load in saliva samples is primarily associated with time from symptom onset and may vary depending on disease severity (12, 20, 21). In the acute phase of the disease (1–10 days after the symptom onset) it can reach up to 1×10^6^–1×10^9^ copies/mL (21, 22). In our study, 61.3% of saliva samples collected from the hospitalized COVID-19 patients were detected as positive with a median viral load of 1 × 10^4.1^ copies/mL, indicating a later stage of the infection and lowered contagiousness, and the positive rRT-PCR test results depended on the time from the symptom onset. The highest virus detection in saliva samples was between 0 and 9 days post-symptom onset, and the finding regarding the median time of 12 days between sample collection and symptom onset for the negative samples was consistent with the reported decrease in viral load.

In our hands, the proposed LFA assay exhibited 26.5% sensitivity and 58.1% specificity in saliva samples collected from the hospitalized COVID-19 patients when compared with rRT-PCR results. Therefore, the minimum requirements for ≥ 90% sensitivity and ≥ 97% specificity defined by the European Commission for a diagnostic test (10) were not achieved. The estimated viral load of 1 × 10^4.1^ copies/mL in saliva samples of the test set was below the limit of detection considered for the antigen assays that could explain the low performance of the developed LFA assay (23).

When specifically targeting patients in the early stage of the SARS-CoV-2 infection by the exclusion of samples that were collected >7 days after the first symptom onset and/or >7 days from laboratory-confirmed diagnosis, there was a major improvement in terms of specificity and PPV of the LFA test. In practice, a substantial improvement in the classification of true positives and true negatives was achieved. The exclusion of samples collected in the later stage of the disease did not reduce the proportion of false-negative results. Therefore, the study results could not be attributed solely to the delayed sample collection, and technical assay improvements are required.

Of note, the universal assay kit used in this study was primarily designed to test selected antibody pairs but not intended to develop a ready-to-use product as the many factors, for example, reagents, gold nanoparticle size, antibody labeling, and conjugate formation protocols, were pre-established by the kit manufacturer. Based on the obtained results, subsequent steps would be an adjustment of reaction conditions to facilitate the formation of the antibody–antigen complex followed by assay validation according to recommendations (10).

As patients are considered contagious if the viral load exceeds 1 × 10^6^ copies/mL, and testing below this threshold does not meet the purpose of the rapid test, a validation procedure should be performed in the relevant population, that is, patients with the asymptomatic and symptomatic forms of the disease in an early stage of infection. In this study, time of the first symptom onset was self-reported; that could introduce a potential source of bias, as a perception of symptoms may differ from patient to patient, thus lacking objectivity.

Another issue to be addressed is the diagnostic performance of the rRT-PCR salivary test itself, as none of the diagnostic tests exhibit 100% sensitivity and specificity. Generally, the false-negative result is multifactorial and could not be underestimated (24). Hence, the use of salivary rRT-PCR as a reference method instead of recommended nasopharyngeal or oropharyngeal swabs could be considered as a limitation of this study (10). The extent of the observed inconsistencies between LFA and rRT-PCR test results that could be explained by the sample type-specific assay performance should be further investigated.

And finally, we used saliva as a diagnostic material for SARS-CoV-2 testing but did not emphasize sample quality depending on the sample collection technique and storage that could potentially have implications on the assay performance.

## Conclusions

Overall, the developed LFA assay demonstrated the potential of detecting the SARS-CoV-2 virus in saliva samples. However, technical improvements should be implemented to achieve excellence in diagnostic performance. A thorough evaluation of the assay following the recommended validation criteria should be performed in a cohort of patients with asymptomatic and symptomatic forms of the disease in the early stage of infection to ascertain the clinical applicability of the proposed test in the target population.
